# Polycystic ovary syndrome and organochlorine pesticides: exploring potential links and mechanisms

**DOI:** 10.3389/frph.2025.1563414

**Published:** 2025-09-02

**Authors:** Shanshan Yin, Wanjia Yang, Feiyun Lin, Mei Jia, Ying Feng, Yanhong Chen, Xiaoxia Bai, Yihan Dong, Shuduan Mao, Kashif Hayat, Xuejing Jin

**Affiliations:** ^1^ Shulan International Medical College, Zhejiang Shuren University, Hangzhou, China; ^2^ The Fourth School of Clinical Medicine, Zhejiang Chinese Medical University, Hangzhou, China; ^3^ Hangzhou Women’s Hospital (Hangzhou Maternity and Child Health Care Hospital), Hangzhou, China; ^4^ Hangzhou Xihu Xixi Community Health Service Center, Hangzhou, China; ^5^ The Affiliated Hospital of Hangzhou Normal University, Hangzhou Normal University, Hangzhou, China; ^6^ Traditional Chinese Medicine for Reproductive Health Key Laboratory of Zhejiang Province, Zhejiang Provincial Clinical Research Center for Obstetrics and Gynecology and Key Lab Womens Reprod Hlth Zhejiang Prov, Women’s Hospital, School of Medicine, Zhejiang University, Hangzhou, China; ^7^ Interdisciplinary Research Academy (IRA), Zhejiang Shuren University, Hangzhou, China

**Keywords:** adrenal glands, hypothalamic-pituitary-ovarian axis, insulin resistance, metabolic abnormalities, organochlorine pesticides, polycystic ovary syndrome

## Abstract

Polycystic ovary syndrome (PCOS) is a prevalent endocrine disorder among women, characterized by metabolic abnormalities and infertility. Despite its high prevalence, the etiology and pathogenesis of PCOS remain poorly understood. Emerging evidence suggests that persistent organic pollutants (POPs), known for their detrimental effects on the endocrine and reproductive systems, may play a role in the development and progression of PCOS. Among POPs, organochlorine pesticides (OCPs) are particularly widespread and pose significant health risks. This review examines the potential of OCPs as an environmental factor in the development and progression of PCOS. It highlights the mechanisms through which OCPs may disrupt the hypothalamic-pituitary-ovarian (HPO) axis and impair hormonal regulation, contributing to the onset and exacerbation of PCOS. Evidence links OCPs to insulin resistance, obesity, and type 2 diabetes mellitus. These disruptions may occur via pathways involving hypothyroidism or altered adrenal androgen secretion. While current evidence supports a plausible connection between OCP exposure and PCOS, significant gaps and inconsistencies in the data warrant further investigation. Elucidating the precise mechanisms underlying these associations is crucial for developing targeted prevention and intervention strategies.

## 1 Introduction

Polycystic ovary syndrome (PCOS) is a prevalent endocrine disorder affecting women of reproductive age and is recognized as the leading cause of infertility. The global prevalence of PCOS ranges from 4% to 21%, varying by diagnostic criteria and population (1). This complex condition is characterized by hyperandrogenism, ovarian dysfunction, and polycystic ovarian morphology. The expression of PCOS is thought to result from a multifactorial etiology involving both genetic and environmental factors (2). Among environmental factors, persistent organic pollutants (POPs) have emerged as potential contributors.

Persistent organic pollutants (POPs) are pervasive environmental contaminants known for bioaccumulation in living organisms. Persistent organic pollutants exhibit bioaccumulation factors >5,000 in adipose tissue (3). A significant proportion function as endocrine-disrupting chemicals (EDCs), interfering with hormone synthesis, secretion, and metabolism. Organochlorine pesticides (OCPs)—a prominent subgroup of POPs—are synthetic chlorine-containing compounds first synthesized in 1874. Historically used as insecticides and for disease control (e.g., malaria), OCPs are highly lipophilic and resistant to biodegradation (4). Due to their persistence and toxicity, global regulatory efforts have targeted OCPs.

The United Nations implemented the Stockholm Convention in 2001 to eliminate POP production and restrict agricultural/industrial use (Stockholm Convention (5). OCPs were included in Annex A given their historical prevalence and environmental persistence (6). OCPs bind to estrogen receptors with affinity constants (K*_d_*) of 10^−8^–10^−9^ M (7), classifying them as prototypical EDCs. Despite regulatory action, the endocrine-disrupting potential of OCPs remains a concern for reproductive disorders like PCOS.

The hypothalamic-pituitary-ovarian (HPO) axis regulates female neuroendocrine function, secretion of luteinizing hormone (LH) and follicle-stimulating hormone (FSH) in the pituitary gland via gonadotropin-releasing hormone (GnRH) secretion from the hypothalamus. Dysregulation of this axis—alongside insulin resistance (IR) -is central to PCOS pathophysiology. There is emerging evidence that serum levels of oral contraceptive pills (OCPs) differ significantly between patients with polycystic ovary syndrome (PCOS) and controls (8–10). This evidence implicates OCPs in reproductive/endocrine disruption. This motivates our systematic examination of OCPs as environmental risk factors.

Therefore, the aim of this review is to: (1) provide a summary of PCOS pathogenesis and clinical manifestations; (2) evaluate the effects of OCPs on reproductive and endocrine systems; (3) analyze the epidemiological and mechanistic links between OCPs and PCOS; and (4) discuss OCPs as potential environmental etiological factors that contribute to the dysregulation associated with PCOS.

## 2 Review strategy

To evaluate the current research perspective on the relationship between PCOS and OCPs, a systematic literature search was conducted using the Web of Science and SCOPUS databases for the period 2004–2024. The search strategy included a combination of topic-specific keywords applied to the title, abstract, and keywords sections of the studies. The search terms included: (i) PCOS, (ii) PCOS and EDC, (iii) OCP, (iv) HPO axis, and (v) endocrine system.

Keywords were as follows:

(i) PCOS: patholo* or mechanism or epidemiolo* or aetiolo*;
(ii) PCOS and EDC: bisphenol A or per- and polyfluoroalkyl substance* or polychlorinated biphenyl*(PCBs) or phthalate* or air pollut* or tributyltin(TBT) or microplastic* or pharmaceuticals and personal care product*(PPCPs) or nanoparticle* or organochlorine pesticide*;
(iii) OCPs: epidemiolog* or soil or plant or food or population risk or estrogen*;
(iv) HPO axis: OCP and hypothalamus or pituitary or ovaries or uterus;
(v) Endocrine system: OCP and insulin resisten* or diabetes or obesity, OCP and thyroid funct*, OCP and adrenal gland or adrenal hormones.

The inclusion criteria were formulated to include articles written in English that explored the pathogenesis, clinical manifestations of PCOS, and the endocrine-disrupting effects of OCPs. While several reviews have examined PCOS from clinical, epidemiological, or pathogenetic perspectives (11–13), this review uniquely emphasizes the potential association between PCOS and a pervasive class of POPs, namely, OCPs. The initial screening process involved reviewing titles and abstracts against the predefined inclusion criteria. Subsequently, full-text articles meeting these criteria were examined in detail. In addition, reference lists of the included articles were manually reviewed for the identification of relevant studies.

## 3 Endocrine disruptors and PCOS: a mechanistic insight

### 3.1 Historical context and diagnostic evolution

PCOS was first described in 1935 by Stein and Leventhal, who observed symptoms including hirsutism, obesity, amenorrhea, and bilaterally enlarged polycystic ovaries (14). Diagnostic criteria have evolved through three major frameworks: the National Institutes of Health (1990), Rotterdam (2003), and Androgen Excess Society (2006) criteria, reflecting ongoing refinement in PCOS characterization (2, 15).

### 3.2 Epidemiological trends and clinical findings

The global prevalence of PCOS ranges from 4% to 21%, varying by diagnostic criteria and population (1). Longitudinal data indicate a rising trend, with the global age-standardized incidence increasing by 1.45% from 2007 to 2017 (reaching 82.44 per 100,000 women) and affecting approximately 66 million women by 2019 (16, 17). Geographical disparities exist, with the highest incidence in Ecuador (242.54 per 100,000) and significant increases in countries with medium-high socio-demographic indices (18).

PCOS typically manifests during puberty, with menstrual dysfunction (e.g., oligomenorrhea, amenorrhea) as the primary feature (19). Clinical hallmarks include hyperandrogenism (hirsutism, acne, alopecia) and metabolic complications, with over 50% of patients exhibiting abdominal obesity (20–22). Notably, IR occurs across BMI categories, affecting a subset of lean patients, while infertility due to anovulation remains a major concern (23, 24).

### 3.3 Core pathophysiological mechanisms

PCOS manifests through three interconnected pathways, summarized in Table 1. The first involves dysregulation of the hypothalamic-pituitary-ovarian (HPO) axis. Increased pituitary sensitivity to gonadotropin-releasing hormone (GnRH) stimulation results in excessive luteinizing hormone (LH) secretion, which promotes androgen production by theca cells and contributes to hyperandrogenemia (25, 26). Concurrently, follicle-stimulating hormone (FSH) levels are relatively low due to negative feedback from estrogen, inhibiting follicular development and leading to a high LH/FSH ratio. This hormonal imbalance further disrupts ovarian follicle maturation and increases anti-Müllerian hormone (AMH) levels (27, 28). Additionally, in a hyperandrogenic environment, the pulsatility of GnRH is impaired, resulting in an increase in LH release and a decrease in FSH release (29).

**Table 1:** Summary of PCOS clinical features and diagnostic criteria.

Domain	Key characteristics	Diagnostic relevance
Reproductive	Oligo/anovulation, infertility, polycystic ovarian morphology	Core features in all criteria
Hyperandrogenism	Clinical (hirsutism, acne) or biochemical (elevated androgens)	Required in NIH/AES; 1 of 3 in Rotterdam
Metabolic	Insulin resistance (50%–75%), obesity (50%–80%), T2DM risk	Not diagnostic but informs management
Phenotype Variability	Lean PCOS (20%–30% of cases) with IR; “non-hyperandrogenic” phenotype (Rotterdam only)	Contributes to heterogeneity

The second key pathway is hyperinsulinemia and insulin resistance (IR). Reduced peripheral insulin sensitivity diminishes its biological efficacy (30). Insulin acts as a potent regulator of androgen production in the ovary, stimulating the growth and secretion of hormones in follicle cells by acting on insulin receptors (31). It also triggers ovarian P450c17 and P450scc enzyme activities to promote ovarian steroidogenesis (32), and in the presence of chorionic gonadotropin, it synergistically increases these activities (33). Excessive insulin levels directly impact pituitary receptors, leading to the release of LH and subsequent stimulation of androgen secretion from the ovaries and adrenal glands. This process increases free testosterone by inhibiting the synthesis of hepatic sex hormone-binding globulin. While IR is a central feature of PCOS (affecting approximately 75% of patients), it is notable that some PCOS patients with normal or low body mass indices develop IR, indicating that the relationship is not absolute (23, 34).

The third pathway encompasses ovarian and endometrial alterations. Compared to women without PCOS, the ovaries of those with the condition are typically bilaterally and uniformly enlarged, ranging from approximately 2–5 times their normal size (35). Additionally, the endometrium in patients with PCOS often exhibits varying degrees of hyperplasia due to estrogen surges associated with prolonged anovulation, which may elevate the risk of developing endometrial cancer (36–38). These pathological changes reflect the systemic impact of hormonal dysregulation on reproductive tissues.

### 3.4 Genetic and environmental etiology

The etiology of PCOS remains incompletely understood but is recognized to involve complex interactions between genetic and environmental factors. From a genetic perspective, the disorder may arise from cumulative effects of polymorphisms in genes related to steroidogenesis, insulin signaling, and chronic inflammation, alongside epigenetic modifications and altered protein profiles that collectively drive systemic dysfunction (39, 40). Environmentally, increasing attention focuses on pollutants, particularly endocrine-disrupting chemicals (EDCs), that can mimic, antagonize, or disrupt hormonal signaling pathways. These compounds may alter normal endocrine homeostasis through multiple mechanisms, as detailed in the following subsection. Of note, lipophilic EDCs like organochlorine pesticides (OCPs) may accumulate in ovarian tissue, potentially exacerbating hormonal dysregulation and contributing to PCOS pathogenesis.

### 3.5 Endocrine-Disrupting chemical actions

Endocrine-disrupting chemicals interfere with hormonal homeostasis through four primary mechanisms (Table 2): (1) mimicking endogenous steroid hormones by binding to nuclear receptors; (2) antagonizing natural hormone actions; (3) altering the synthesis, metabolism, or clearance of endogenous hormones; and (4) modifying hormone receptor expression in target tissues (41). A growing body of evidence suggests correlations between PCOS and exposure to environmental contaminants acting through these pathways. Notably, OCPs—as prototypical EDCs—exemplify these mechanisms by binding estrogen receptors and inducing estrogen-like responses, thereby disrupting reproductive and metabolic functions relevant to PCOS.

**Table 2:** EDC mechanisms relevant to PCOS pathways.

EDC action	Compounds	Potential PCOS impact
Estrogen receptor agonism	*o,p*′*-*DDT, BPA, methoxychlor	HPO axis disruption; aberrant folliculogenesis
Androgen receptor antagonism	*p,p*′*-*DDE, vinclozolin	Hyperandrogenism via feedback dysregulation
Steroidogenic enzyme alteration	PFAS, phthalates	Altered ovarian/adrenal androgen output
Insulin signaling disruption	PCBs, BPA	Exacerbation of IR

## 4 Environmental exposure to EDCs and PCOS

### 4.1 Evidence for key EDC classes

EDCs encompass diverse substances including pesticides, industrial chemicals, plasticizers, and pharmaceuticals that are ubiquitous in modern environments (42). These compounds interfere with sex steroid hormone synthesis, action, and metabolism, contributing to adverse reproductive outcomes including developmental toxicity, infertility, and hormone-related cancers (3). There have also been reports of interference with the hypothalamic-pituitary-thyroid and adrenal axes (43). Four major classes show documented associations with PCOS pathogenesis through distinct pathways (Table 3):

**Table 3:** Evidence from previous studies on the possible correlation of EDCs with PCOS.

EDC	Industrial or commercial application	Possible correlation to PCOS	References
Bisphenol A	Plastic bottles, dental fillings, and the lining of food cans	Increasing secretion of matrix metalloproteinase-9, activating the receptor to downregulate glucose transporters-4, expression and increasing metabolic risk promote PCOS.	(44–46)
Per- and polyfluoroalkyl substances	Everyday products, e.g., food packaging, and cloth coating	Increasing risk of developing PCOS, especially in overweight women.	(47–49)
Polychlorinated biphenyls	Dielectric fluids and hydraulic fluids	Potential action on microRNAs, is more pronounced in individuals with higher body mass indices	(50, 51)
Di (2-Ethylhexyl) Phthalate	Plasticizers	An increased likelihood of developing PCOS	(52, 53)
Tributyltin	Plastic preparations and pharmaceutical intermediates	Induce phenotypic production of PCOS	(54)
Pharmaceuticals and personal care products	Fragrances, cosmetics, sunscreens, hair dyes, prescription and over-the-counter medications	Induce metabolic dysfunction, presence of estrogen effects	(55)
Organochlorine pesticides	Pesticides	Affecting hormonal levels in the development of PCOS	(9, 10)

#### 4.1.1 Bisphenol A (BPA)

As the most extensively studied EDC in PCOS contexts, BPA exhibits mild estrogenic and anti-androgenic properties. Human biomonitoring reveals urinary BPA correlates with elevated testosterone in PCOS patients (56), while *in vitro* studies demonstrate it increases matrix metalloproteinase-9 secretion in granulosa cells (44). Proposed mechanisms include downregulation of glucose transporter-4 via aryl hydrocarbon receptor activation (46) and multigenerational effects evidenced in medaka fish models (57). BPA exposure is associated with visceral obesity, hyperinsulinemia, dyslipidemia, and elevated androgens in PCOS patients (45). While human evidence is robust (*n* = 1,046 in Milanovic et al.), causal inference is limited by predominantly cross-sectional designs.

#### 4.1.2 Per/Polyfluoroalkyl Substances (PFAS)

PFAS (“forever chemicals”) used in textiles, food packaging, and firefighting foams demonstrate environmental persistence (47). Elevated PFAS levels associate with increased PCOS risk (48, 58), particularly among overweight/obese women. Recent analyses confirm PFAS as significant contributors to PCOS risk profiles (49). Mechanistic evidence remains less developed than for BPA, though obesogenic effects are well-documented.

#### 4.1.3 Polychlorinated Biphenyls (PCBs)

These dielectric fluids accumulate in adipose tissue due to extended half-lives. PCBs have been linked to PCOS through microRNA interactions (50), with stronger correlations observed in higher-BMI individuals (131). Counterevidence exists regarding menstrual cycle hormone impacts (59). Discrepant findings highlight phenotype-specific susceptibility and exposure timing variables.

#### 4.1.4 Phthalates and PPCPs

Di(2-ethylhexyl) phthalate (DEHP) exposure correlates with increased PCOS likelihood (52), with mixture exposure showing stronger effects in urinary metabolite analyses (53). Environmentally obesogenic tributyltin induces PCOS-like reproductive, metabolic, and cardiovascular abnormalities (54), while select pharmaceuticals and personal care products (PPCPs) disrupt metabolic function through estrogenic, anti-estrogenic, and anti-androgenic activities (55). PPCP evidence derives primarily from animal models, warranting human cohort validation.

#### 4.1.5 Organochlorine Pesticides (OCPs)

Emerging human evidence shows higher serum OCP levels (*p,p*′*-*DDE, *o,p*′*-*DDT) in PCOS patients vs. controls (9, 10). This supports OCPs as environmental contributors to PCOS pathogenesis, though mechanistic research remains limited compared to other EDCs.

### 4.2 Human exposure to OCPs and EDC effects

Human exposure to organochlorine pesticides occurs primarily through inhalation of contaminated air, ingestion of tainted food and water sources, and direct dermal contact with polluted environmental media, as evidenced by biomonitoring studies across diverse populations (60). Global biomonitoring data confirm widespread body burden, with breast milk samples from four Asian countries showing 100% detection rates for *p,p*′*-*DDT and *p,p*′*-*DDE (61). In the United States, the National Health and Nutrition Examination Survey (NHANES) reported mean serum *p,p*′*-*DDE concentrations of 238 ng/g lipid during 2003–2004 (62), while contemporary Chinese cohorts exhibit particularly elevated levels, including pentachlorophenol concentrations reaching 3.13 μg/L in Beijing residents (51). Australian longitudinal data demonstrate temporal declines across five sampling periods (2002–2013), though persistent detection confirms ongoing exposure despite regulatory restrictions (63).

OCPs exert significant adverse effects on female reproductive development and function across the lifespan. Adolescent exposure correlates with delayed physical and sexual maturation (64), while reproductive-age women experience dose-dependent ovarian impacts including follicular atresia, altered steroid secretion patterns, and diminished oocyte viability (65). Transplacental transfer occurs consistently across populations, with maternal serum concentrations exceeding fetal levels despite partial placental barrier function (66), as demonstrated in Wuhan cohort studies where median hexachlorocyclohexane (HCH) and DDT concentrations in umbilical cord serum reached 10.1 ng/g lipid and 35.5 ng/g lipid, respectively (67).

Molecular mechanisms underlying these effects involve multifaceted endocrine disruption pathways. OCPs elicit estrogenic responses through direct binding to estrogen receptors (7) while simultaneously modulating steroidogenesis via transcriptional regulation of aromatase (CYP19A1) in granulosa cells (68). Androgen receptor antagonism has been documented in receptor binding assays (69), and early developmental pathway alterations occur through epigenetic reprogramming effects observed in fish models (70). In a separate study, *p,p*′*-*DDE inhibited 17 alpha-methyltestosterone (TES)-induced sult2st3 expression in zebrafish embryos; however, at higher concentrations of TES, *p,p*′*-*DDE did not inhibit sult2st3 induction but rather enhanced TES-induced sult2st3 expression (71). These findings collectively demonstrate that OCPs disrupt reproductive physiology through receptor-mediated signaling and enzymatic interference, contributing to PCOS-relevant endocrine dysfunction.

## 5 Associations between OCPs and the HPO axis

**Figure 1:**
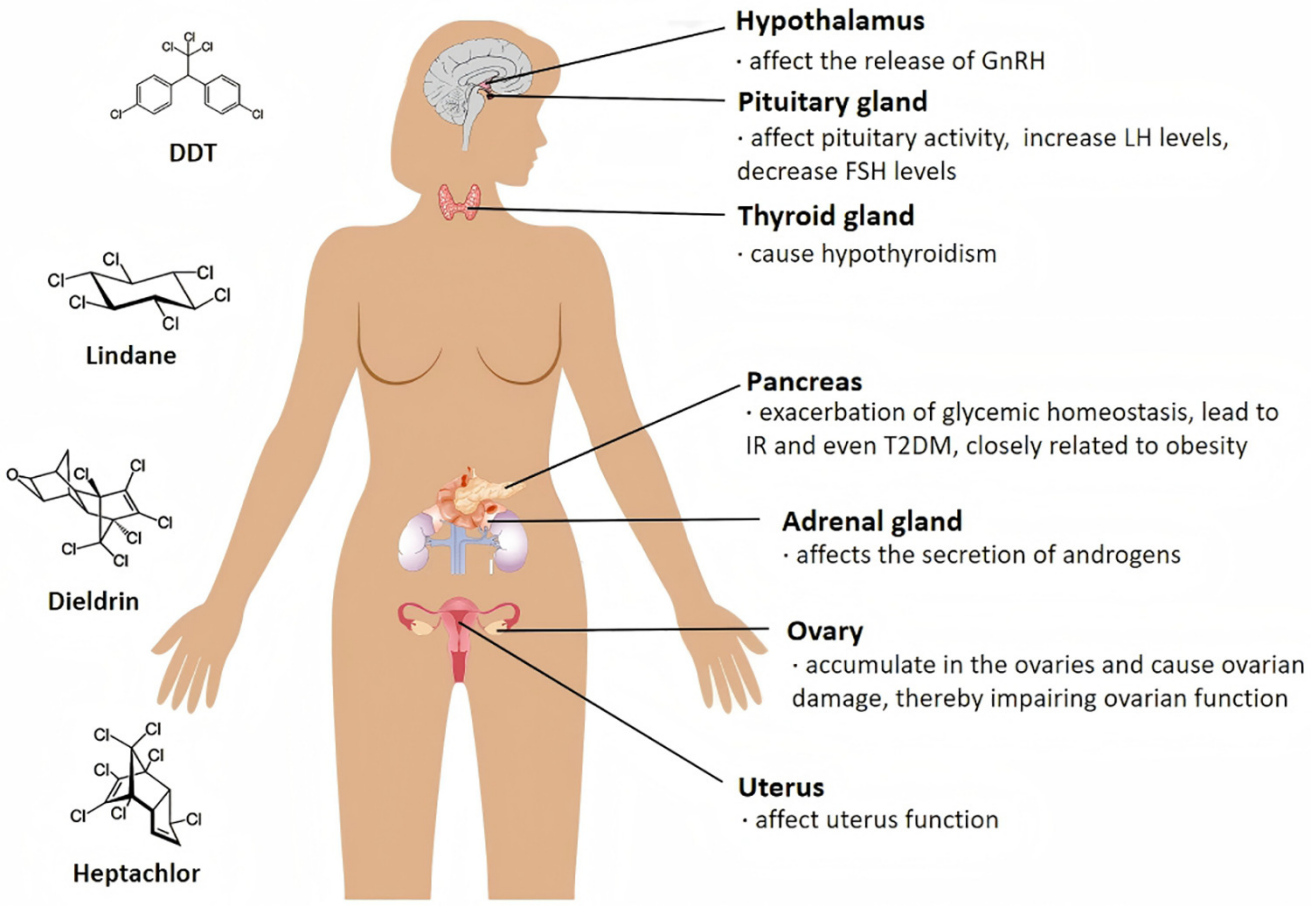
Proposed mechanisms linking environmental EDCs to PCOS development. The schematic diagram represents a hypothetical illustration of the potential adverse effects of OCPs on female internal organs. Notably, the diagram does not represent definitive conclusions but rather provides a hypothetical representation of the potential impact of these compounds.

The hypothalamic-pituitary-ovarian (HPO) axis represents a primary target for OCP-induced endocrine disruption, as illustrated in Figure 1. Evidence suggests OCPs impair regulatory functions at three anatomical levels:

### 5.1 Hypothalamic-pituitary dysregulation by OCPs

Previous reports have suggested that exposure to OCPs during neurodevelopment is associated with altered cytokine homeostasis in the hypothalamus, which would favor changes in hormonal communication.

*In vitro* studies revealed that metolachlor increased GnRH mRNA levels in GT1-7 cells, whereas another study revealed that it reduced them in female mice, with reduced ESR2 (ERβ) expression in the medial preoptic region and no significant change in ESR1 (ERα) expression (72). The results from other studies have also indicated that methoxychlor is associated with an increase in kisspeptin cell expression in the anterior ventral periventricular region of the medial preoptic area, whereas chronic dieldrin exposure alters hypothalamic messenger RNA and protein abundance (73). Endosulfan reduces catfish tryptophan hydroxylase 2 (tph2) and gonadotropin-releasing hormone (cfGnRH) transcript levels and significantly reduces forebrain/preoptic tph2 immunoreactivity (74).

The results from *in vivo* studies further confirmed that some OCPs, e.g., methoxychlor, have a direct effect on the pituitary gland (75). Moreover, *o,p*′*-*DDD, methoxychlor, and HCH can stimulate the growth of the rat pituitary cell line MtT/E-2 both *In vitro* and *in vivo* (132). Endosulfan may act as a downstream mediator of LH receptor activation and as an upstream mediator of the steroidogenic enzymes studied, leading to reduced pituitary FSH levels (37). Endosulfan may affect pituitary activity by inducing the gene expression of nitric oxide synthase in rats. It increases plasma LH and GH levels but decreases plasma thyroid-stimulating hormone concentrations (76).

In a highly polluted area in Brazil, human biomonitoring studies have suggested that organochlorine pesticides are negatively correlated with LH and FSH in perimenopausal and menopausal women (77).

### 5.2 Ovarian-uterine pathological effects of OCPs

A study of stranded pregnant sperm whales revealed that contaminants such as DDT and HCH were present at higher concentrations in the ovaries than in the epidermis. OCPs may accumulate in ovarian tissue through metabolism and trigger adverse effects (78). *In vitro* studies suggest that β*-*HCH, DDE, and dieldrin induce reactive oxygen species, proinflammatory responses, and DNA damage in human ovarian surface epithelial cells (79). Chlordane and endrin stimulate the secretion of testosterone and estradiol from the granulosa cells of the ovaries of cows, whereas toxaphene and heptachlor can inhibit this effect (80, 81). Pentachloronitrobenzene can alter MAPK3/1 signaling and progesterone production and accelerate follicular development while increasing AMH in ovarian tissue and serum (82). HCB inhibits lipofuscin secretion and expression in follicle cells by directly inhibiting lipofuscin (83) and decreases lipocalin-stimulated E2 secretion, which may contribute to ovarian dysfunction in obesity-related diseases. Human ovarian cells exposed to the HCB and *p,p*′*-*DDE presented a decrease in the proportion of unilamellar follicles, an increase in follicular atresia, and altered expression of LDHA, ATP5A and GPX4 in exposed tissues, as well as altered ATP production in KGN and tissue cultures.

The results from animal studies also indicated that OCPs could affect ovarian/uterus function. Methoxychlor causes follicular atresia by inducing apoptosis in granulosa cells (84), which can damage ovarian epithelial cells and oocytes (85) and reduce serum estradiol and progesterone levels (86). It can also alter the expression of genes related to intracellular and membrane trafficking, intra-ovarian signaling, and intercellular junctions and communication in piglet ovaries (87). Early methoxychlor exposure may result in accelerated initial recruitment of ovarian follicles and impaired cyclic recruitment of sinusoidal follicles. It has also been proposed that both methoxychlor and its metabolite, 2,2-bis(4-hydroxyphenyl)-1,1,1-trichloroethane, could inhibit early ovarian development and stimulate AMH production by granulosa cells in the rat ovary. These effects may further reduce estrogen production and reduce estrogen synthesis through the inhibition of FSH-stimulated estrogen receptors, thereby reducing cAMP production (88). Early methoxychlor exposure may result in accelerated initial recruitment of ovarian follicles and impaired cyclic recruitment of sinusoidal follicles (89), while it has also been proposed that both methoxychlor and its major metabolite inhibit early ovarian development and stimulate AMH production by granulosa cells in the rat ovary (90), which may reduce estrogen synthesis by inhibiting FSH-stimulated estrogen receptors (ESR1 and 2), thereby reducing cAMP production. Dieldrin affects the reproductive endocrine system in buffalo granulosa cells (68) by regulating the proximal promoter, leading to increased specific CYP19A1 transcription and elevated estrogen levels. Lindane (91) can increase the level of the Cx43 gap junction blocker lindane in mouse granulosa cells, leading to ultrastructural damage and apoptosis.

High levels of OCPs in the follicular fluid (92) adversely affect the outcome of intracytoplasmic sperm injection. The higher the OCP concentration measured in samples of human origin was, the lower the sampling rate, fertilization rate and embryo retrieval rate were. Human biomonitoring studies have detected OCPs in ovarian fluid samples from female populations. In 127 follicular fluid samples from randomly selected fertile women in China, 17 OCPs were detected, with chloromethyl chloride being the most common, followed by heptachlor epoxide, hexachlorocyclohexane, endrin, and DDT (93). In Uppsala, Sweden, OCPs were found in both blood and follicular fluid samples collected from 185 women (94). Current knowledge suggests that OCPs have the capacity to disrupt physiological function through exposure, but the response‒dose relationships between OCPs and adverse health outcomes warrant further investigation.

## 6 Associations between OCPs and the endocrine system

### 6.1 Associations between OCPs and IR and obesity

Insulin, which is secreted by beta cells in the pancreas, works with glucagon to regulate blood glucose levels (95). It induces glucose storage in the liver, muscles and adipose tissue, leading to weight gain (96, 97). IR is physiologically defined as a state of reduced responsiveness of insulin target tissues to high physiological levels of insulin (98). IR is one of the major symptoms of PCOS, as approximately 75% of patients with PCOS will develop it (99), and a higher percentage of patients with PCOS who are obese will suffer from IR (100).

Insulin has two main pathways. The metabolic pathway is mainly mediated by phosphatidylinositol 3-kinase and protein kinase B, also known as the phosphatidylinositol 3-kinase pathway. The second pathway is the mitogenic pathway, which refers to the mitogen-activated protein kinase-extracellular signal-regulated kinase pathway. Increased serine phosphorylation and decreased tyrosine phosphorylation of the insulin receptor and IRS terminate insulin action, leading to insulin dysfunction in women with PCOS (100, 101).

OCPs can lead to IR and even diabetes. Exposure to lindane (102) and DDT (103) at sub-toxic concentrations could induce oxidative stress in mouse myotubular cells, activating receptor-sensitive kinases, impairing insulin signaling pathways and promoting IR effects. An *In vitro* study revealed that *p,p*′*-*DDE causes pancreatic islet beta cells to become dysfunctional and destroyed (104). Exposure to DDE alters the plasma lipid metabolomic profile while impairing glucose homeostasis and even inducing dysbiosis of the gut flora (105). *p,p*′*-*DDE may play a role in the regulation of immune cell function, leading to immune system dysfunction resulting in IR or metabolic syndromes (106).

Studies suggest that human exposure to OCPs may cause biological, metabolic, and endocrine disruption. Reports from cross-sectional studies have shown a positive correlation between OCP and the risk of metabolic syndromes, such as abdominal obesity and dysglycemia (51, 107–109). Longitudinal studies have also shown that long-term exposure to OCPs could worsen people's glycemic homeostasis (110, 111) and increase the risk of metabolic syndrome (111, 112). Serum OCP concentrations play an important role in the development of human metabolic phenotypes, and this conclusion also applies to healthy individuals of normal weight (113, 114). The vast majority of PCOS patients have abdominal obesity and IR, leading to the hypothesis that OCP accumulation is more likely in PCOS patients than in the general population and that OCP may be one of the environmental factors contributing to PCOS patients with IR or obesity.

A strong association between exposure to OCPs and type 2 diabetes mellitus (T2DM) has been reported in epidemiological studies. For example, a study in northern India reported significantly higher levels of β-HCH, dieldrin and *p,p*′*-*DDE in a pre-diabetic group and a newly identified diabetic group than in a normal population (115). Serum concentrations of OCPs are significantly associated with the risk of T2DM in adults in Saudi Arabia exposed to OCPs (116). The risk of T2DM was found to be greatly increased in people exposed to higher levels of OCPs via contaminated drinking water (117) and/or living environments (118). OCPs, such as the HCBs in adipose tissue or fat, have also been reported to be significantly associated with IR and diabetes (119).

OCPs are also associated with obesity, another major symptom of PCOS. The results from animal studies have shown that Endrin promotes early adipogenesis in 3T3-L1 cells through the activation of mammalian target of rapamycin (120). Another study revealed that exposure to DDE increased body weight and adiposity in mice (105). *P,p*′*-*DDE was also found to be able to induce the expression of macrophage surface markers in mouse adipose tissue and affect macrophage reactivity *In vitro*, thereby promoting fat synthesis (106). Similar findings have been reported in human biomonitoring. The results of a cross-sectional study revealed that waist circumference was strongly associated with OCPs in 117 patients who underwent non oncological surgery (109). OCPs may also be linked to the worsening of obesity-related clinical complications (121). There was a strong correlation between OCPs and two of the characteristic symptoms of PCOS, namely, IR and obesity. However, the underlying mechanisms of these effects require further *in vitro, in vivo* and *in silico* studies.

### 6.2 OCP and the thyroid gland

There is evidence to suggest that an increased incidence of thyroid disorders, including autoimmune thyroiditis and subclinical hypothyroidism, is associated with PCOS (122). Animal studies have suggested that pre-pregnancy exposure to HCB causes hypothyroidism, which has serious implications for female reproduction by severely affecting the functional connectivity of the thyroid and ovaries (123), and a study in Belgium revealed that residual organochlorine contaminants in umbilical cord blood were associated with thyroid hormones in newborns and the risk of maternal hypothyroidism (124). Existing studies suggest a link between thyroid disease and PCOS, but the causal relationship is unclear, and more research is needed (122).

### 6.3 OCP and adrenal glands

The adrenal glands can secrete androgens, and excessive secretion can cause PCOS in women. A possible link between OCPs and the adrenal glands is therefore also worthy of investigation. Animal studies have shown that methoxychlor reduces adrenal secretion of DHEA, StAR, CYP11A1, and HSD3B while increasing the expression of CYP17A1 and SULT2A1; i.e., OCPs may downregulate genes involved in the first step of adrenal androgen synthesis (125). Methoxychlor increased the number of fetal testicular mesenchymal stromal cells and stimulated CYP11A1 expression in mice exposed *in utero*. However, testosterone production was inhibited at high doses of methoxychlor (126). Disruption of the secretory function of chromaffin cells in the adrenal gland by prenatal and postnatal exposure to DDT in male Wistar rats (127). Studies suggest that paternal exposure to *p,p*′*-*DDE may have some effect on the offspring of male rats (128).

A biomonitoring study in humans revealed that dieldrin is negatively associated with serum testosterone levels in a Chinese male population, whereas *p,p*′*-*DDD is positively associated with serum testosterone levels (129). The effects of OCPs on the adrenal glands and their possible associations with PCOS warrant further investigation.

### 6.4 Genetic susceptibility modifiers

While studies directly linking detoxification SNPs to OCP metabolism in PCOS remain limited, preliminary evidence suggests genetic variants may modulate susceptibility. For example, CYP1A1 polymorphisms are associated with altered organochlorine pesticide clearance in general populations (130), though PCOS-specific pharmacogenomic investigations are scarce. Future studies should prioritize genotyping in well-characterized PCOS cohorts with OCP exposure biomarkers. This represents a critical research frontier, understanding how genetic variation in detox pathways (e.g., GST deletions, CYP polymorphisms) interacts with OCP accumulation to potentially amplify PCOS risk requires targeted investigation.

## 7 Current knowledge gaps and perspectives

### 7.1 Limitations

The following limitations must be considered when interpreting the findings of this review: Firstly, the heterogeneity in PCOS diagnostic criteria (Rotterdam vs. NIH) complicates cross-study comparisons. Secondly, there is a clear geographical bias in favour of data on Asian and European exposure, with limited representation of the Global South. Thirdly and finally, causal inference in this domain is constrained by the preponderance of cross-sectional human studies. To advance our understanding, it is essential to employ longitudinal designs that systematically track the accumulation of OCPs in the period preceding the onset of PCOS.

### 7.2 Research gaps and perspectives

PCOS is a prevalent condition that affects women's health and is increasingly recognized as a significant public health concern, with its incidence rising each year. There is growing evidence that OCP may be detrimental to the female reproductive system and could be one of the environmental factors contributing to the etiology of PCOS.

Available literature suggests that OCPs may affect the HPO axis by affecting the hypothalamus and pituitary gland. This could lead to elevated production of GnRH and LH, alongside a decrease in FSH, resulting in endocrine disruption within the HPO axis. However, studies examining the effects of OCPs on the hypothalamus and pituitary are limited and often yield conflicting results, highlighting a significant gap in our understanding.

Furthermore, it has been suggested that OCPs can accumulate in the ovaries and cause ovarian damage, impairing ovarian function. These chemicals may also contribute to endocrine dysregulation among women, potentially causing or exacerbating the onset and progression of PCOS. Additionally, there is a possibility that some OCPS could lead to hypothyroidism and influence androgen production by the adrenal glands. While it is conceivable that various OCPs may have cumulative effects on ovarian function, the precise mechanisms by which specific OCPs or combinations of them affect the ovaries remain unclear. The existing evidence regarding the effects of OCPs on thyroid and adrenal function is inconsistent, indicating that further research is essential to elucidate these mechanisms.
